# Supplementary material for: Multiple dynamic interactions from basal ganglia direct and indirect pathways mediate action selection
Source: bioRxiv. 2023 Mar 21:2023.03.20.533567. Preprint. [Version 1] doi: 10.1101/2023.03.20.533567 (PMC10055198; doi:10.1101/2023.03.20.533567)
Supplement: Supplement 1 [file NIHPP2023.03.20.533567v1-supplement-1.pdf]

bioRxiv preprint doi: https://doi.org/10.1101/2023.03.20.533567; this version posted March 21, 2023. The copyright holder for this preprint (which was not certified by peer review) is the author/funder, who has granted bioRxiv a license to display the preprint in perpetuity. It is made available under aCC-BY 4.0 International license.

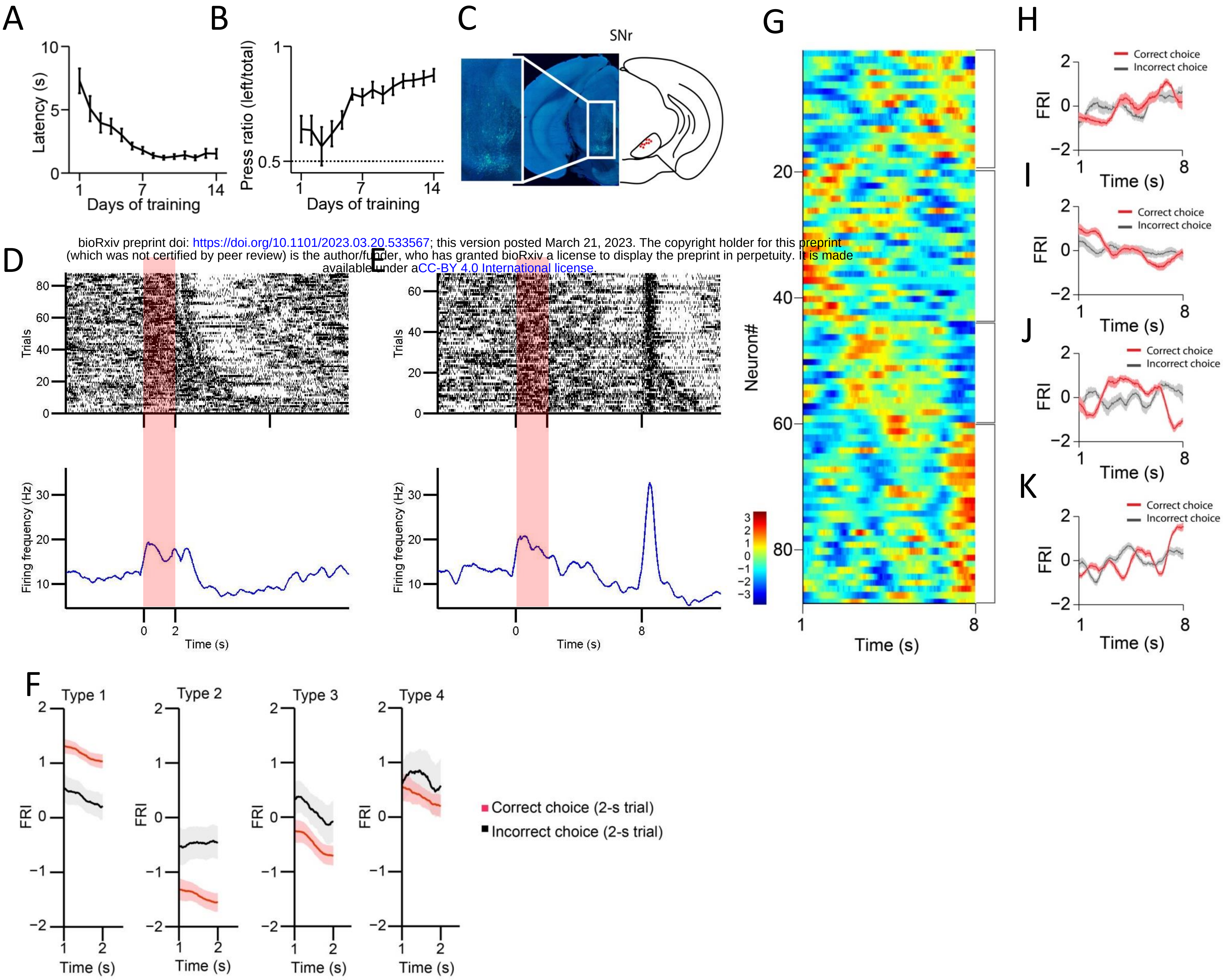

Figure S1. Behavioral performance across 14 days of training and the SNr neuronal recording on day 1. (A) Lever press latency after lever extension for wild type mice across 14 days training (n = 10 mice, one-way repeated-measures ANOVA, effect of training days, $F_{13,117} = 21.32$, $p < 0.0001$). (B) Lever press ratio for wild type mice across 14 days training (n = 10 mice, one-way repeated-measures ANOVA, effect of training days, $F_{13,117} = 6.472$, $p < 0.0001$). (C) Example of recording array placement in SNr (left) and validation of array placement in a cohort of wildtype mice (right). Inset better demonstrates small tracts formed by the array implant. (D, E) Firing activities of an example SNr neuron in correct 2-s (D) and 8-s trials (E) after 14 days training. Top panels: raster plot of spikes across trials aligned to lever retraction at time 0. Bottom panel: PETH plot. Red shaded areas highlight the initial 2-s period after the lever retraction in 2-s (D) and 8-s trials (E). (F) Averaged FRI for Type 1, Type 2, Type 3 and Type 4 of SNr neurons in correct (red) and incorrect 2-s trials (gray). (G) Firing Rate Index (FRI) of neuronal activity for all task-related SNr neurons in correct 8-s trials on day 1 of training. The magnitude of FRI is color coded and the SNr neurons are categorized as four subgroups based on the activity dynamics. (H-K) Averaged FRI of SNr neurons in correct (red) and incorrect 8-s trials (gray) on day 1 of training.

bioRxiv preprint doi: https://doi.org/10.1101/2023.03.20.533567; this version posted March 21, 2023. The copyright holder for this preprint (which was not certified by peer review) is the author/funder, who has granted bioRxiv a license to display the preprint in perpetuity. It is made available under aCC-BY 4.0 International license.

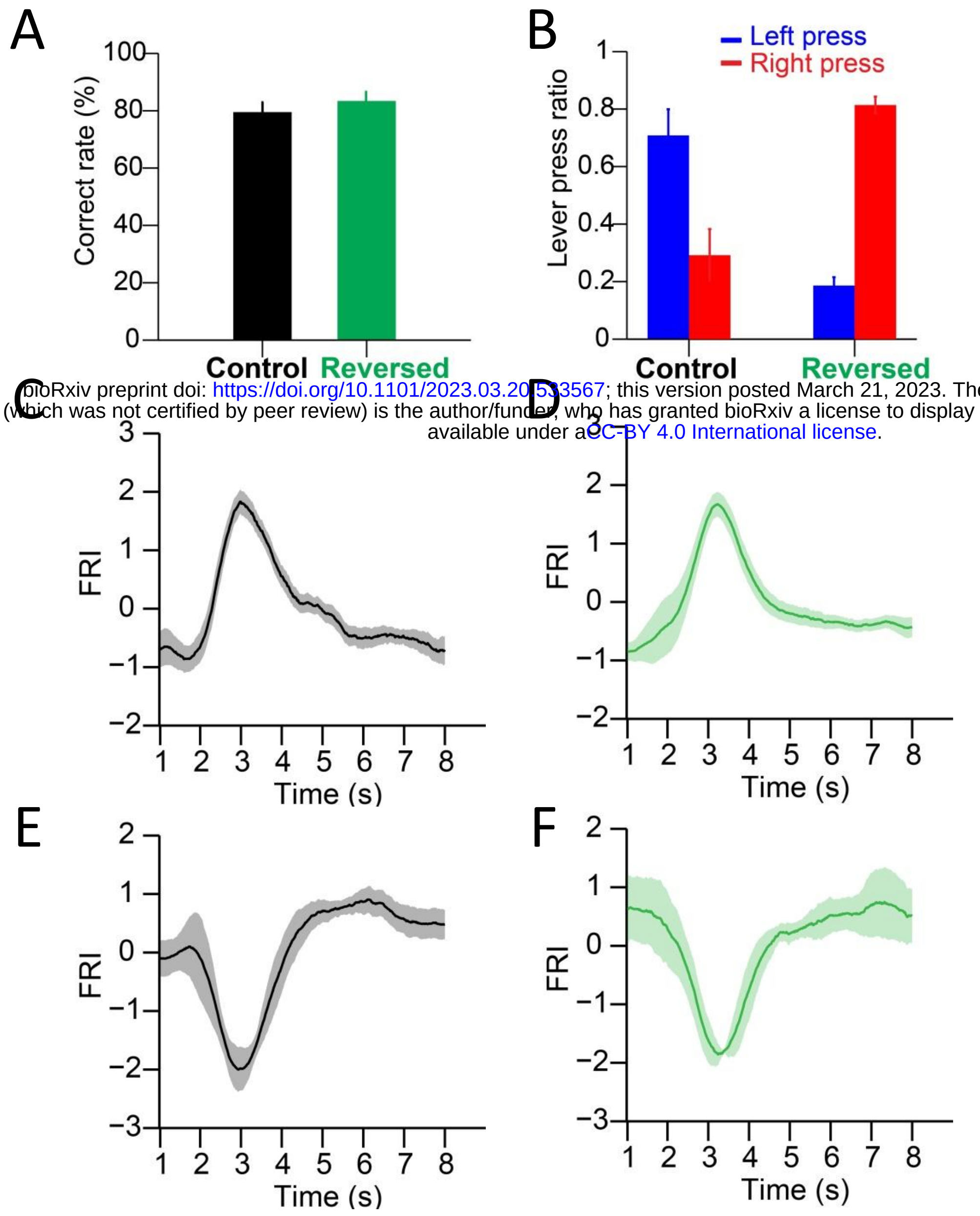

Figure S2. Behavioral statistics and neuronal dynamics of SNr neurons in the standard and reversed 2-8s tasks . (A) Correct rates of the same group of mice both in the standard and reversed 2-8s tasks (n = 6 mice, paired t-test, $p = 0.33$). (B) Lever press ratios of the same group of mice both in the standard and reversed 2-8s tasks (n = 6 mice, paired t-test, $p < 0.05$). (C) Averaged FRI of the SNr Type 3 neurons in correct 8-s trials of the standard 2-8s task. (D) Averaged FRI of the SNr Type 3 neurons in correct 8-s trials of the reversed 2-8s task. (E) Averaged FRI of the SNr Type 4 neurons in correct 8-s trials of the standard 2-8s task. (F) Averaged FRI of the SNr Type 4 neurons in correct 8-s trials of the reversed 2-8s task.

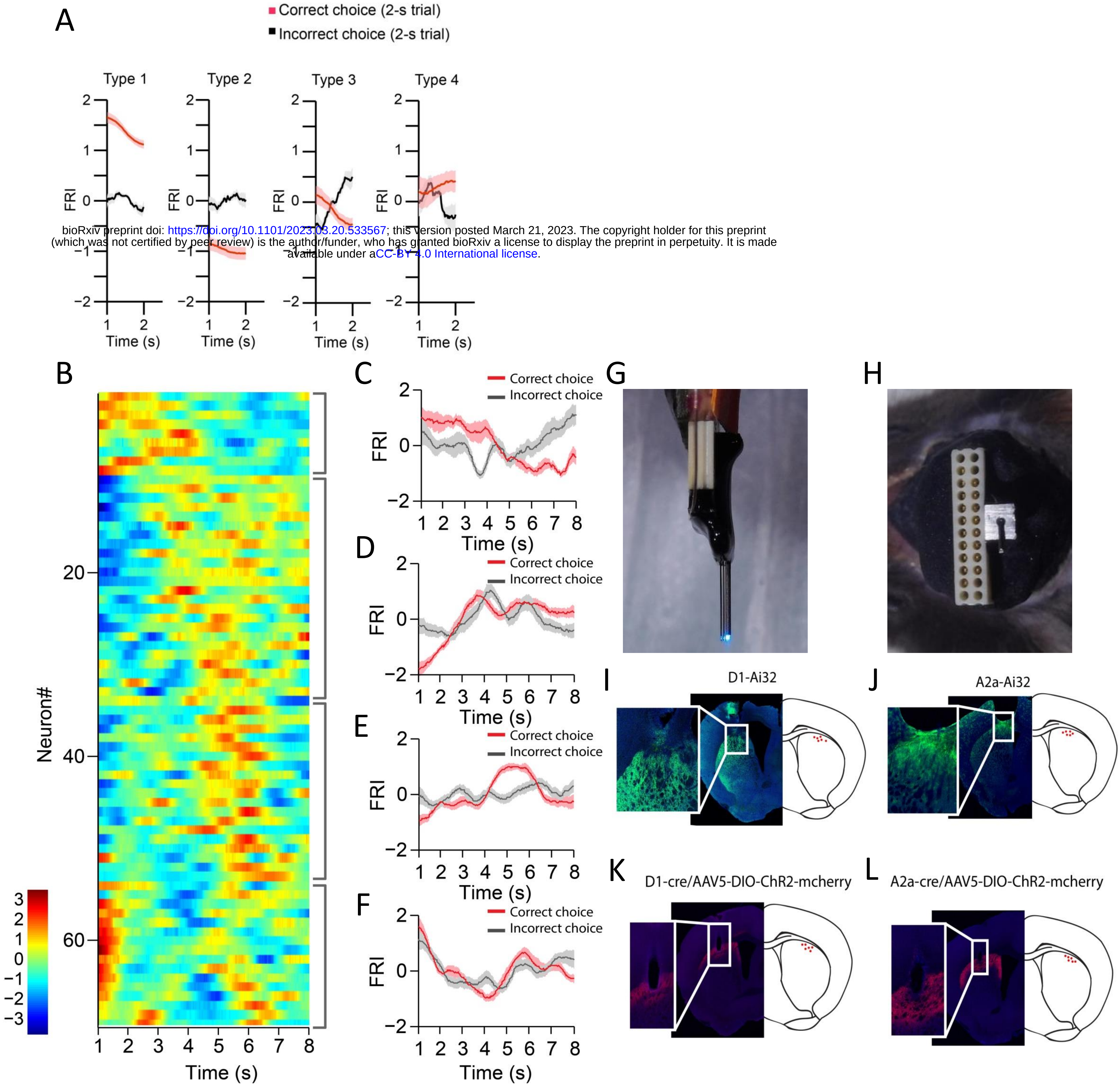

bioRxiv preprint doi: https://doi.org/10.1101/2023.03.20.533567; this version posted March 21, 2023. The copyright holder for this preprint (which was not certified by peer review) is the author/funder, who has granted bioRxiv a license to display the preprint in perpetuity. It is made available under aCC-BY 4.0 International license.

Figure S3. Striatum neuronal recording on day 1 of training, recording array and optic fiber placement validation. (A) Averaged FRI for Type 1, Type 2, Type 3 and Type 4 of SPNs in correct (red) and incorrect 2-s trials (gray). (B) Firing Rate Index (FRI) of neuronal activity for all task-related SPNs in correct 8-s trials on day 1 of training. The magnitude of FRI is color coded and the SPNs are categorized as four subgroups based on the activity dynamics. (C-F) Averaged FRI of SPNs in correct (red) and incorrect 8-s trials (gray). (G) Recording array affixed with a cannula implanted in D1-Ai32 or A2a-Ai32 mice. Light emitted by optic fiber placed through the attached cannula is in close proximity to the tips of the recording array. (H) Top-down view of the array implantation. (I) Example of array placement in dorsal striatum of a D1-Ai32 mouse (left) and validation of fiber placement in a cohort of D1-Ai32 mice (right). Inset better demonstrates the tract formed by the array implant. (J) Example of array placement in dorsal striatum of a A2a-Ai32 mouse (left) and validation of fiber placement in a cohort of A2a-Ai32 mice (right). Inset better demonstrates the tract formed by the array implant. (K) Example of fiber placement in dorsal striatum of a D1-cre mouse with AAV5-DIO-ChR2-mcherry injected (left) and validation of fiber placement in a cohort of D1-cre mice (right). Inset better demonstrates the tract formed by the fiber implant. (L) Example of fiber placement in dorsal striatum of a A2a-cre mouse with AAV5-DIO-ChR2-mcherry injected (left) and validation of fiber placement in a cohort of A2a-cre mice (right). Inset better demonstrates the tract formed by the fiber implant.

bioRxiv preprint doi: https://doi.org/10.1101/2023.03.20.533567; this version posted March 21, 2023. The copyright holder for this preprint
(which was not certified by peer review) is the author/funder, who has granted bioRxiv a license to display the preprint in perpetuity. It is made
available under aCC-BY 4.0 International license.

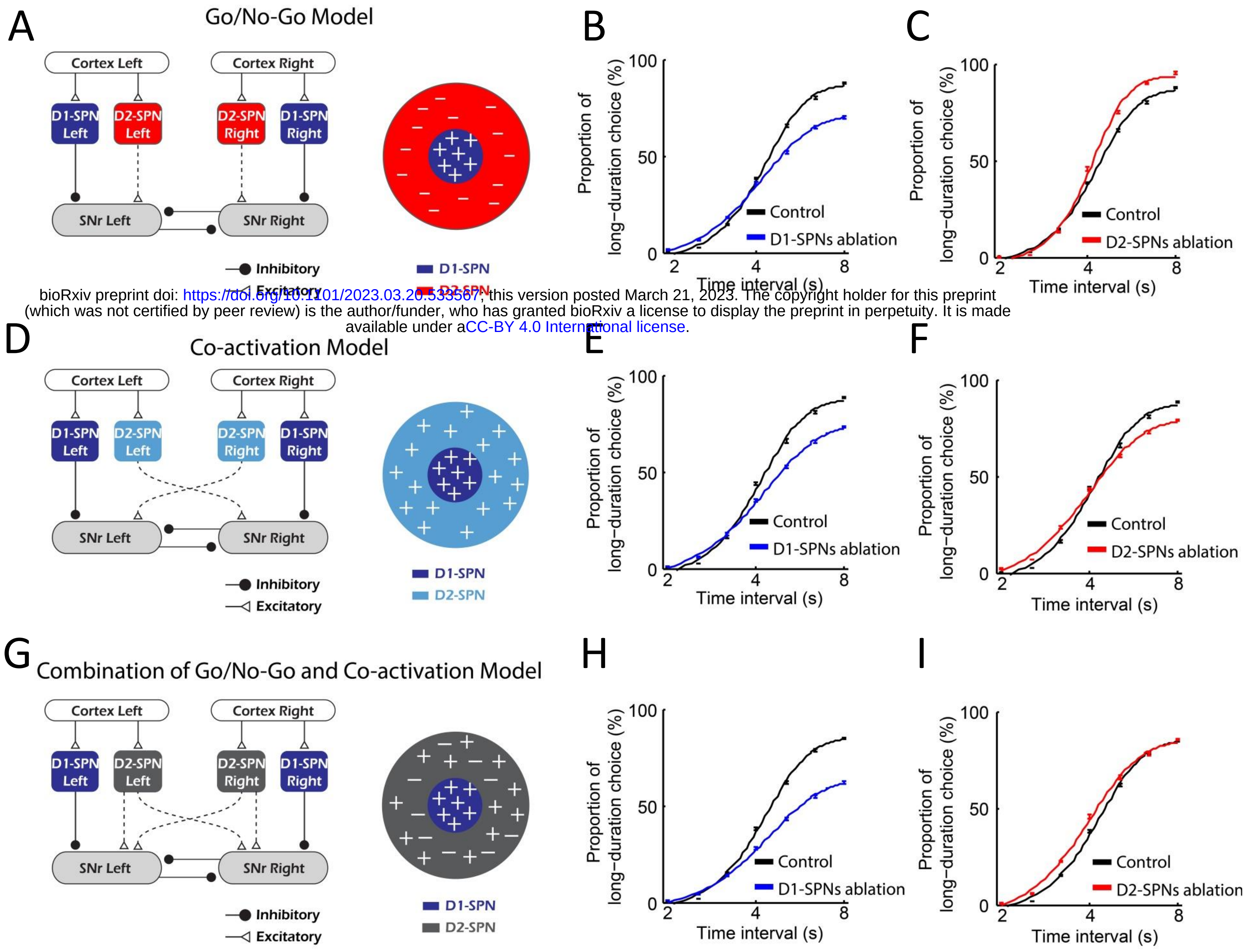

Figure S4. Simulation of lesion experiments in Go/No-Go, Co-activation and combination models. (A) Diagram of Go/No-Go model. (B) The psychometric curves of behavior outputs simulated by Go/No-Go model in control (black) and D1-SPNs ablation condition (blue). (C) The psychometric curves of behavior outputs simulated by Go/No-Go model in control (black) and D2-SPNs ablation condition (red). (D) Diagram of Co-activation model. (E) The psychometric curves of behavior outputs simulated by Co-activation model in control (black) and D1-SPNs ablation condition (blue). (F) The psychometric curves of behavior outputs simulated by Co-activation model in control (black) and D2-SPNs ablation condition (red). (G) Diagram of combination of Go/No-Go and Co-activation model. (H) The psychometric curves of behavior outputs simulated by combined model in control (black) and D1-SPNs ablation condition (blue). (I) The psychometric curves of behavior outputs simulated by combined model in control (black) and D2-SPNs ablation condition (red).

bioRxiv preprint doi: https://doi.org/10.1101/2023.03.20.533567; this version posted March 21, 2023. The copyright holder for this preprint (which was not certified by peer review) is the author/funder, who has granted bioRxiv a license to display the preprint in perpetuity. It is made available under aCC-BY 4.0 International license.

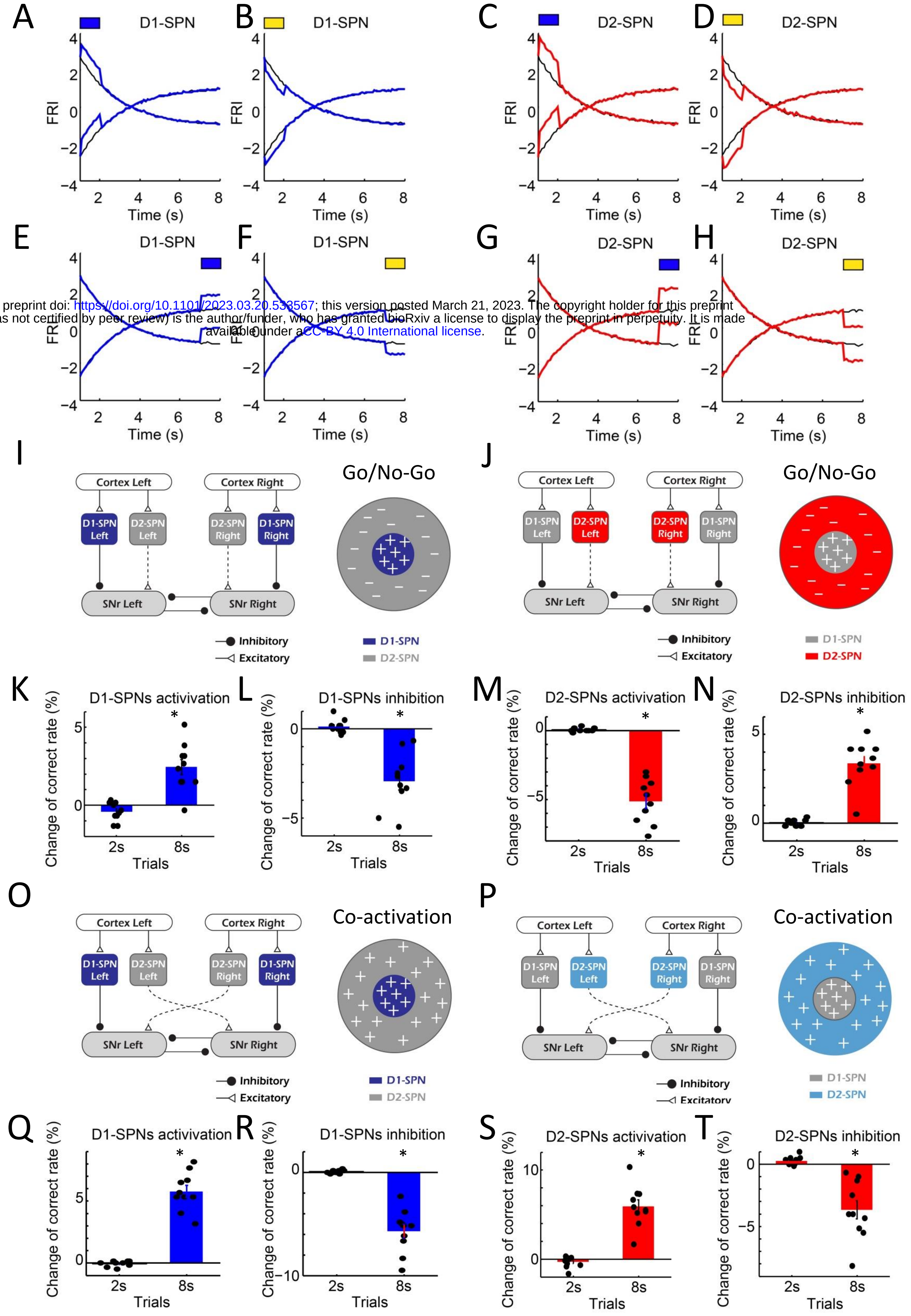

Figure S5. Simulation of optogenetic manipulation in Go/No-Go and Co-activation models. (A, B) Simulating optogenetic activation (A) and inhibition (B) of D1-SPNs at 2s. Blue bar above indicates optogenetic activation. Yellow bar above indicates optogenetic inhibition. (C, D) Simulating optogenetic activation (C) and inhibition (D) of D2-SPNs at 2s. (E, F) Simulating optogenetic activation (E) and inhibition (F) of D1-SPNs at 8s. (G, H) Simulating optogenetic activation (G) and inhibition (H) of D2-SPNs at 8s. (I, J) Diagram of D1-SPN (I) and D2-SPN (J) manipulation in Go/No-Go model. (K, L) Change of correct rate in 2-s and 8-s trials when activating (K) and inhibiting (L) D1-SPNs in Go/No-Go model. (M, N) Change of correct rate in 2-s and 8-s trials when activating (M) and inhibiting (N) D2-SPNs in Go/No-Go model. (O, P) Diagram of D1-SPN (O) and D2-SPN (P) manipulation in Co-activation model. (Q, R) Change of correct rate in 2-s and 8-s trials when activating (Q) and inhibiting (R) D1-SPNs in Co-activation model. (S, T) Change of correct rate in 2-s and 8-s trials when activating (S) and inhibiting (T) D2-SPNs in Co-activation model. One-sample test for all the change of correct rate. *$p < 0.05$.

bioRxiv preprint doi: https://doi.org/10.1101/2023.03.20.533567; this version posted March 21, 2023. The copyright holder for this preprint (which was not certified by peer review) is the author/funder, who has granted bioRxiv a license to display the preprint in perpetuity. It is made available under aCC-BY 4.0 International license.

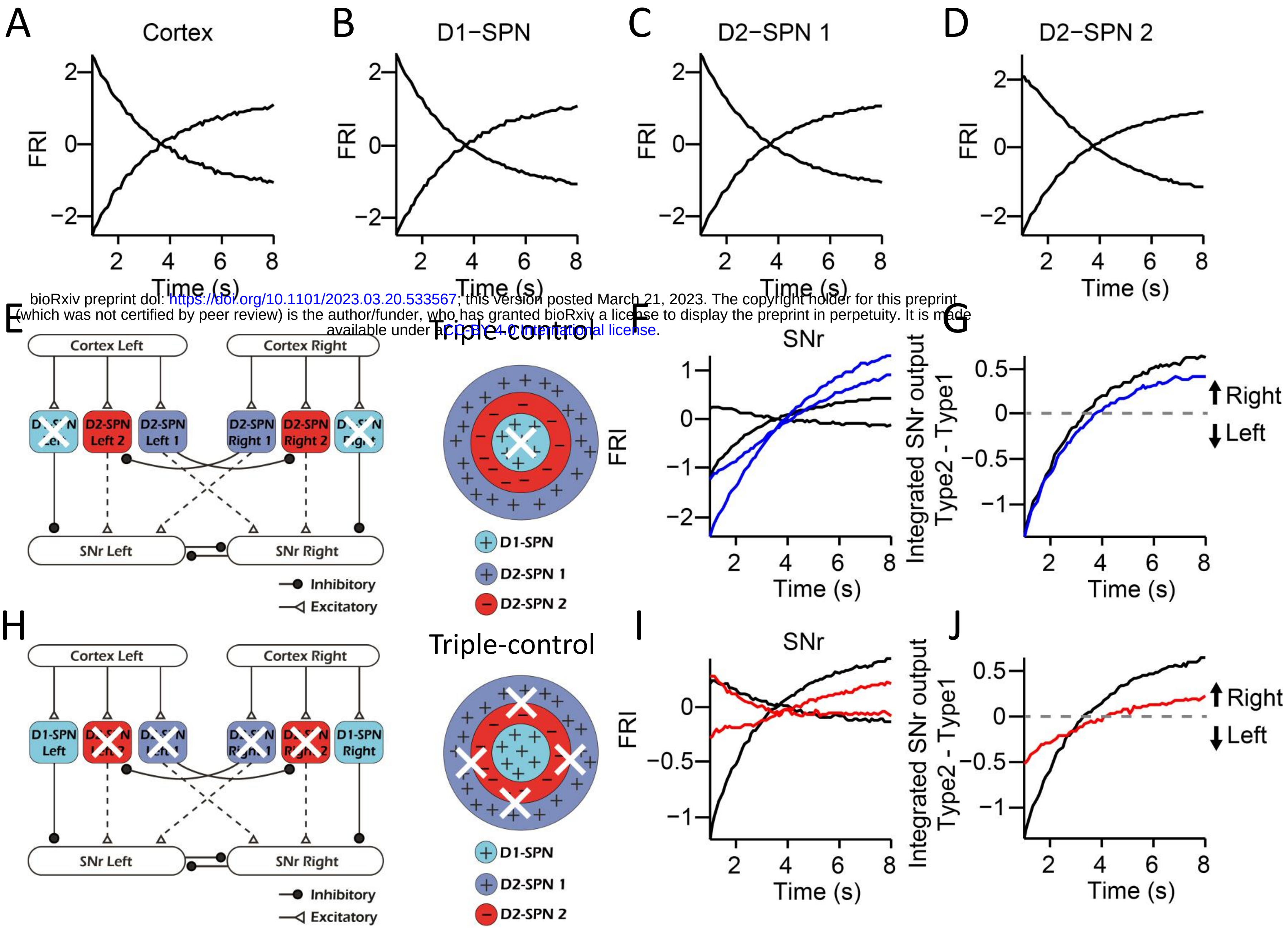

Figure S6. The neuronal activities in the "Triple-control" model and simulation of lesion experiments. (A) The simulated neuronal dynamics quantified as FRI for the cortical neurons in 8s trials. (B) The simulated neuronal dynamics quantified as FRI for the D1-SPN in 8s trials. (C) The simulated neuronal dynamics quantified as FRI for the D2-SPN 1 in 8s trials. (D) The simulated neuronal dynamics quantified as FRI for the D2-SPN 2 in 8s trials. (E) Schematic of selective ablation of D1-SPNs in the "Triple-control" model. (F) The model's Type 1 and Type 2 SNr FRI in control condition (black) and under D1-SPNs ablation (blue). (G) The subtraction of FRI between Type 1 and Type 2 SNr neurons in control (black) and D1-SPNs ablation condition (blue). (H) Schematic of selective ablation of D2-SPNs in the "Triple-control" model. (I) The model's Type 1 and Type 2 SNr FRI in control condition (black) and under D2-SPNs ablation (red). (J) The subtraction of FRI between Type 1 and Type 2 SNr neurons in control (black) and D2-SPNs ablation condition (red).

bioRxiv preprint doi: https://doi.org/10.1101/2023.03.20.533567; this version posted March 21, 2023. The copyright holder for this preprint (which was not certified by peer review) is the author/funder, who has granted bioRxiv a license to display the preprint in perpetuity. It is made available under aCC-BY 4.0 International license.

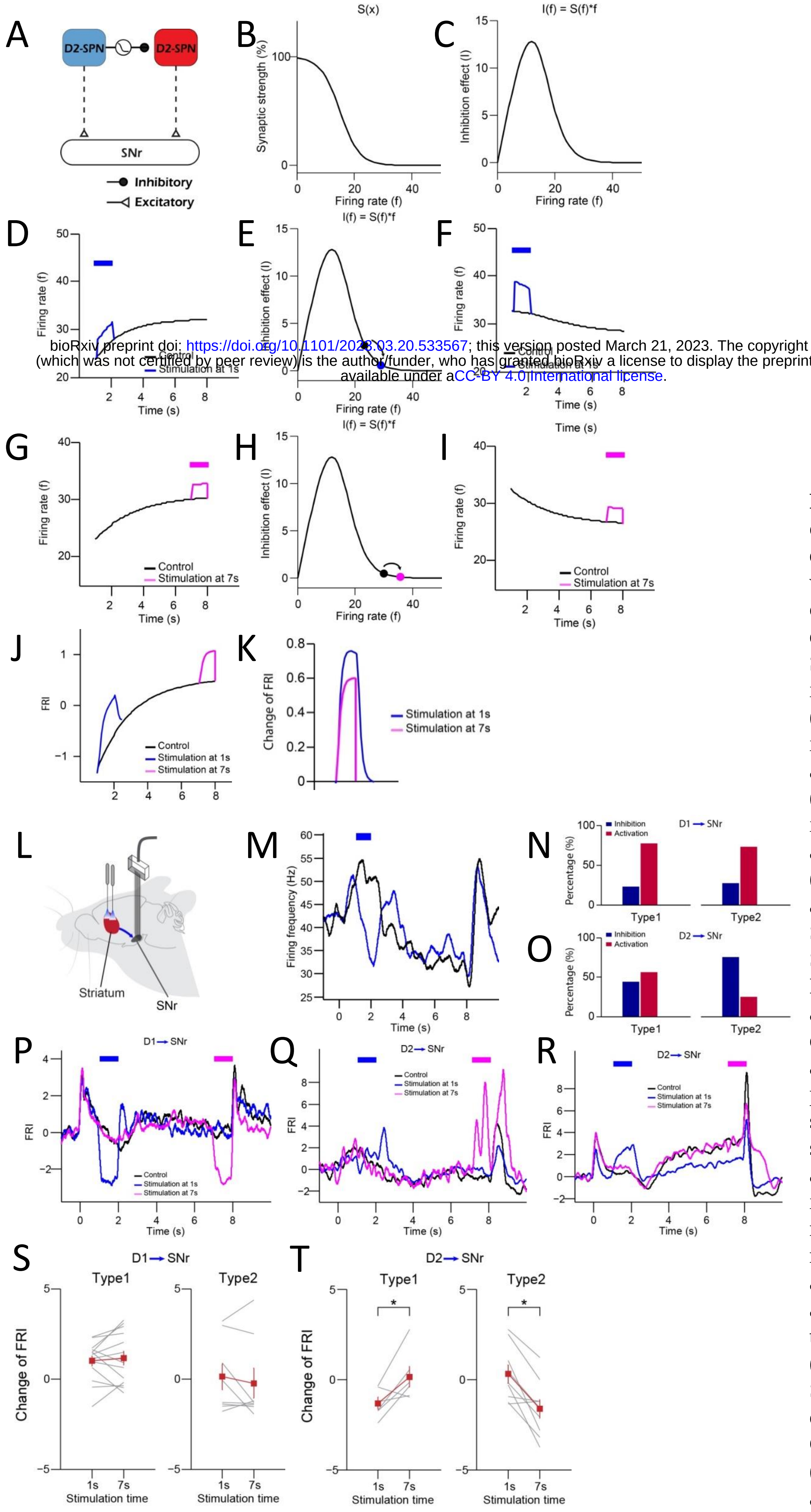

Figure S7. Optogenetic activation of D1- vs. D2-SPNs differently regulates SNr activities in model and experiments. (A) A computational motif of indirect pathway with collateral inhibitory synapse D2-SPN 1→ D2-SPN 2. The collateral synapse between D2-SPNs exhibits short-term depression. (B) Relationship between synaptic strength of D2-SPN 1→ D2-SPN 2 and the firing rate of D2-SPN 1. (C) The inhibition effect of the collateral synapse between D2-SPNs. (D) Activation of presynaptic D2-SPN 1 at 1s. (E) Synaptic inhibition effect D2-SPN 1→ D2-SPN 2 synapse when activating D2-SPN 1 at 1s. (F) Activation of D2-SPN 2 at 1s. (G) Activation of presynaptic D2-SPN 1 at 7s. (H) Synaptic inhibition effect D2-SPN 1→ D2-SPN 2 synapse when activating D2-SPN 1 at 7s. (I) Activation of D2-SPN 2 at 7s. (J) SNr neuron activities responding to activation of D2-SPNs at 1s (blue) and 7s (purple). (K) Comparison of FRI changes in SNr caused by activation of D2-SPNs at 1s and 7s. (L) Schematic of simultaneous optogenetic excitation of D1- or D2-SPNs in the dorsal striatum and recording in SNr during action selection. (M) Averaged neuronal activities of an example SNr Type 1 neuron responding to optogenetic activation of D1-SPNs at 1s during 8-s trials. (N) The percentage of SNr Type 1 (left) and Type 2 (right) neurons showing excitation (blue) and inhibition (red) when stimulating D1-SPNs. (O) The percentage of SNr Type 1 (left) and Type 2 (right) neurons showing excitation (blue) and inhibition (red) when stimulating D2-SPNs. (P) Averaged neuronal activities of an example SNr Type 2 neuron responding to optogenetic activation of D1-SPNs at 1s (blue) and 8s (purple) during 8-s trials. (Q, R) Averaged neuronal activities of SNr Type 1 (Q) and Type 2 (R) neuron responding to optogenetic activation of D2-SPNs at 1s (blue) and 7s (purple) during 8-s trials. (S) Comparison of FRI changes in SNr Type 1 (left) and Type 2 (right) neurons caused by optogenetic activation of D1-SPNs at 1s and 7s. (T) Comparison of FRI changes in SNr Type 1 (left) and Type 2 (right) neurons caused by optogenetic activation of D2-SPNs at 1s and 7s (paired t-test, $p < 0.05$).

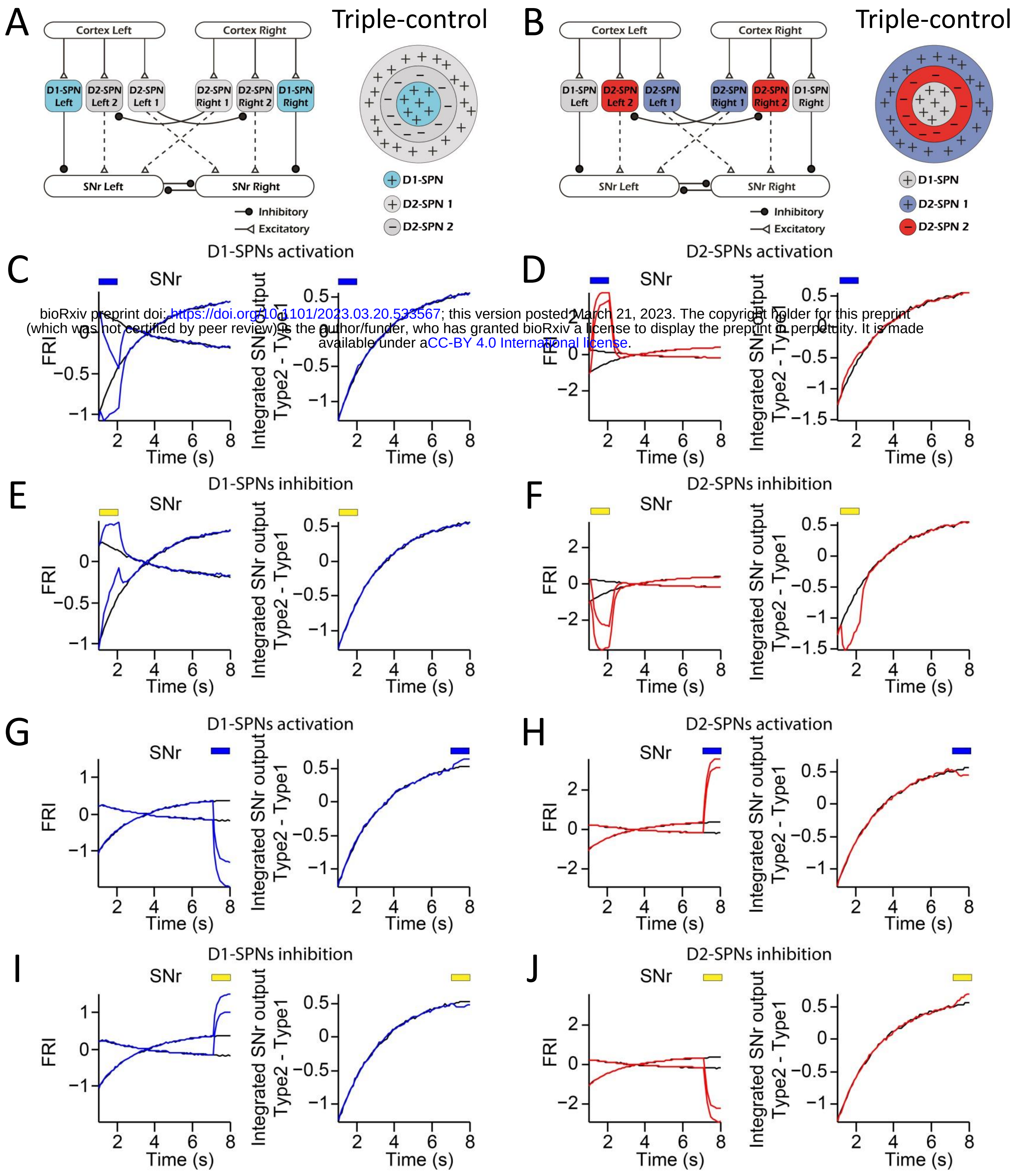

bioRxiv preprint doi: https://doi.org/10.1101/2023.03.20.533567; this version posted March 21, 2023. The copyright holder for this preprint (which was not certified by peer review) is the author/funder, who has granted bioRxiv a license to display the preprint in perpetuity. It is made available under aCC-BY 4.0 International license.

Figure S8. Computational modeling of optogenetic manipulation reveals that D1- vs. D2-SPNs differently regulates SNr outputs in the "Triple-control" model. (A, B) Schematic for optogenetic manipulation of D1-SPNs (A) and D2-SPNs (B) in the 'Triple-control' model. (C) Modeling of neuronal dynamics of SNr Type 1/Type 2 (left panel) and integrated output (right panel) under control (black) and activation (blue) of D1-SPNs at 2s. (D) Modeling of neuronal dynamics of SNr Type 1/Type 2 (left panel) and integrated output (right panel) under control (black) and activation (red) of D2-SPNs at 2s. (E) Modeling of neuronal dynamics of SNr Type 1/Type 2 (left panel) and integrated output (right panel) under control (black) and inhibition (blue) of D1-SPNs at 2s. (F) Modeling of neuronal dynamics of SNr Type 1/Type 2 (left panel) and integrated output (right panel) under control (black) and inhibition (red) of D2-SPNs at 2s. (G) Modeling of neuronal dynamics of SNr Type 1/Type 2 (left panel) and integrated output (right panel) under control (black) and activation (blue) of D1-SPNs at 8s. (H) Modeling of neuronal dynamics of SNr Type 1/Type 2 (left panel) and integrated output (right panel) under control (black) and activation (red) of D2-SPNs at 8s. (I) Modeling of neuronal dynamics of SNr Type 1/Type 2 (left panel) and integrated output (right panel) under control (black) and inhibition (blue) of D1-SPNs at 8s. (J) Modeling of neuronal dynamics of SNr Type 1/Type 2 (left panel) and integrated output (right panel) under control (black) and inhibition (red) of D2-SPNs at 8s.

bioRxiv preprint doi: https://doi.org/10.1101/2023.03.20.533567; this version posted March 21, 2023. The copyright holder for this preprint (which was not certified by peer review) is the author/funder, who has granted bioRxiv a license to display the preprint in perpetuity. It is made available under aCC-BY 4.0 International license.

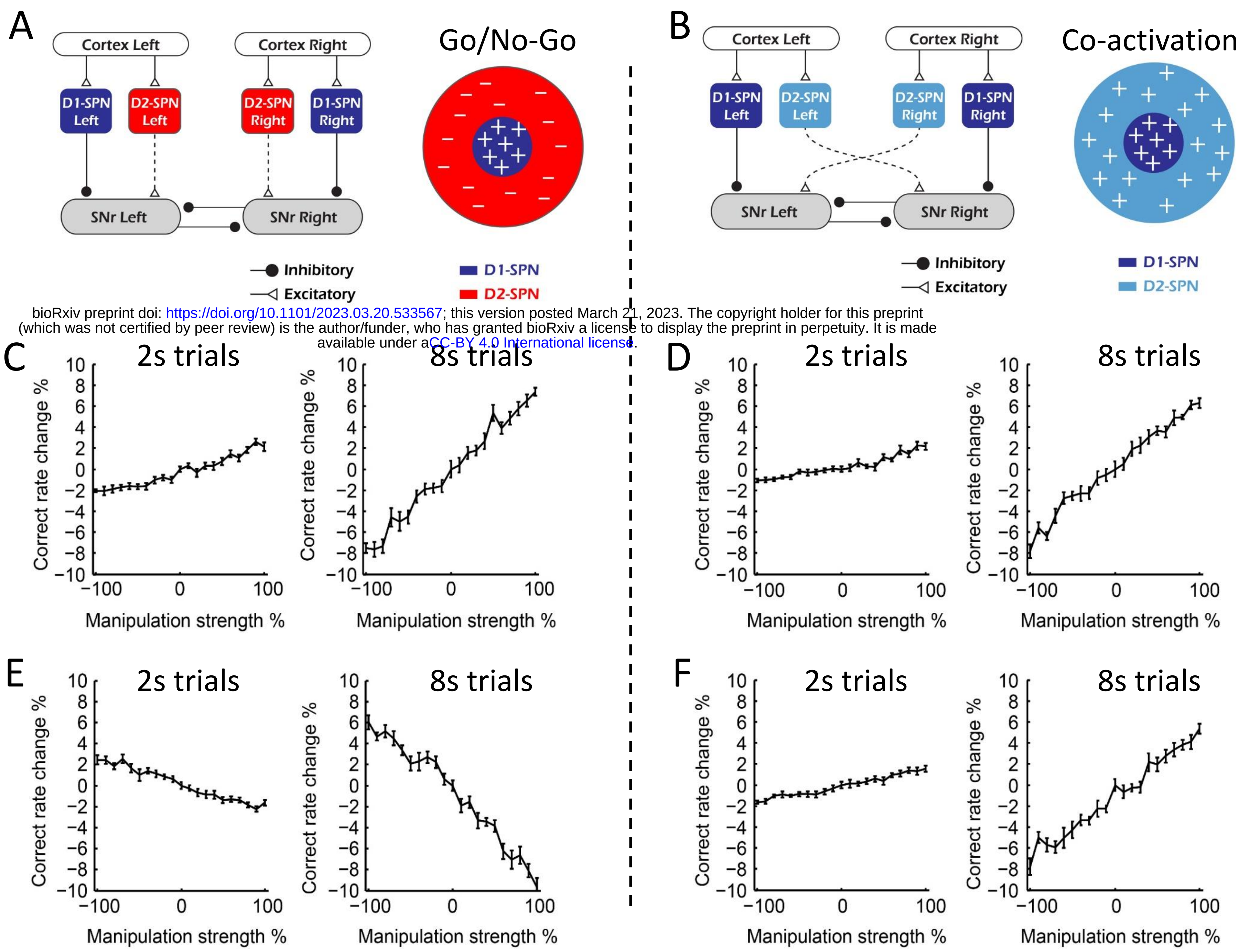

Figure S9. Computational modeling of manipulation reveals that Go/No-Go and Co-activation model differently predicts the behavioral outcomes. (A) Diagram of Go/No-Go model. (B) Diagram of Co-activation model. (C) Correct rate change in 2s (left panel) and 8s trials (right panel) when manipulating D1-SPNs in Go/No-Go model with different manipulation strengths. (D) Correct rate change in 2s (left panel) and 8s trials (right panel) trials when manipulating D1-SPNs in Co-activation model with different manipulation strengths. (E) Correct rate change in 2s (left panel) and 8s trials (right panel) when manipulating D2-SPNs in Go/No-Go model with different manipulation strengths. (F) Correct rate change in 2s (left panel) and 8s trials (right panel) trials when manipulating D2-SPNs in Co-activation model with different manipulation strengths.

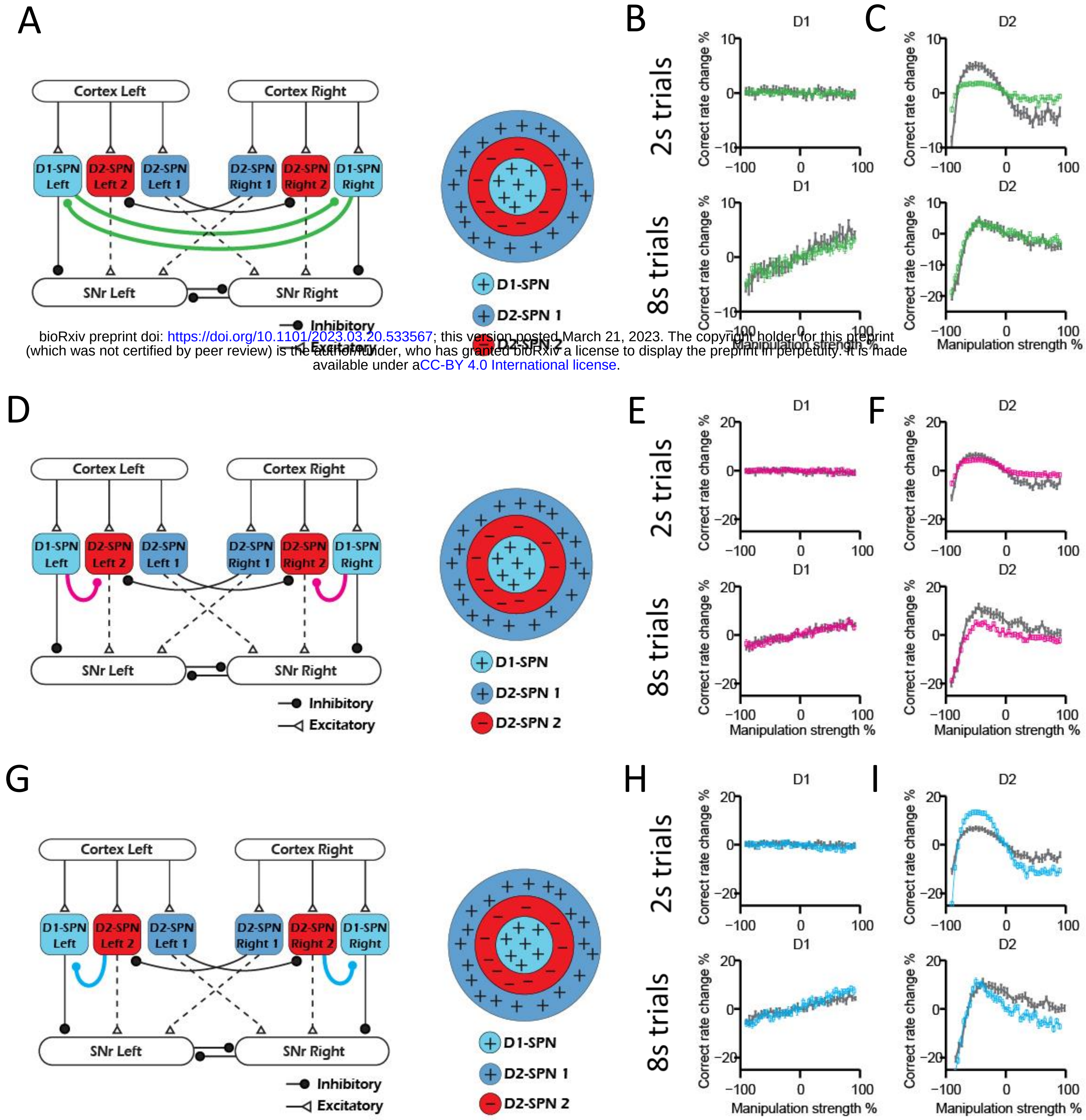

bioRxiv preprint doi: https://doi.org/10.1101/2023.03.20.533567; this version posted March 21, 2023. The copyright holder for this preprint (which was not certified by peer review) is the author/funder, who has granted bioRxiv a license to display the preprint in perpetuity. It is made available under aCC-BY 4.0 International license.

Figure S10. Computational modeling reveals that the linear and nonlinear modulation of action selection by direct versus indirect pathway qualitatively hold with additional striatal collateral connections. (A) Schematic for 'Triple-control' model with D1-D1 collateral connections. (B) Correct rate change in 2s trials (upper panel) and 8s trials (bottom panel) when manipulating D1-SPNs with different manipulation strengths (n = 10, one-way repeated-measures ANOVA, effect of manipulation strength, 2s trials: $F_{40,369} = 1.328$, $p = 0.0945$; 8s trials: $F_{40,369} = 7.595$, $p < 0.0001$). For comparison, the same simulation results as in Figure 7(C, G) are shown in gray. (C) Correct rate change in 2s trials (upper panel) and 8s trials (bottom panel) when manipulating D2-SPNs with different manipulation strengths (n = 10, one-way repeated-measures ANOVA, effect of manipulation strength, 2s trials: $F_{40,369} = 38.22$, $p < 0.0001$; 8s trials: $F_{40,369} = 34.29$, $p < 0.0001$). For comparison, the same simulation results as in Figure 7(D, H) are shown in gray. (D) Schematic for 'Triple-control' model with D1-D2 collateral connections. (E) Correct rate change in 2s trials (upper panel) and 8s trials (bottom panel) when manipulating D1-SPNs with different manipulation strengths (n = 10, one-way repeated-measures ANOVA, effect of manipulation strength, 2s trials: $F_{40,369} = 0.9335$, $p = 0.5893$; 8s trials: $F_{40,369} = 8.778$, $p < 0.0001$). For comparison, the same simulation results as in Figure 7(C, G) are shown in gray. (F) Correct rate change in 2s trials (upper panel) and 8s trials (bottom panel) when manipulating D2-SPNs with different manipulation strengths (n = 10, one-way repeated-measures ANOVA, effect of manipulation strength, 2s trials: $F_{40,369} = 40.94$, $p < 0.0001$; 8s trials: $F_{40,369} = 26.61$, $p < 0.0001$). For comparison, the same simulation results as in Figure 7(D, H) are shown in gray. (G) Schematic for 'Triple-control' model with D2-D1 collateral connections. (H) Correct rate change in 2s trials (upper panel) and 8s trials (bottom panel) when manipulating D1-SPNs with different manipulation strengths (n = 10, one-way repeated-measures ANOVA, effect of manipulation strength, 2s trials: $F_{40,369} = 0.6827$, $p = 0.9299$; 8s trials: $F_{40,369} = 10.06$, $p < 0.0001$). For comparison, the same simulation results as in Figure 7(C, G) are shown in gray. (I) Correct rate change in 2s trials (upper panel) and 8s trials (bottom panel) when manipulating D2-SPNs with different manipulation strengths (n = 10, one-way repeated-measures ANOVA, effect of manipulation strength, 2s trials: $F_{40,369} = 153.3$, $p < 0.0001$; 8s trials: $F_{40,369} = 38.38$, $p < 0.0001$). For comparison, the same simulation results as in Figure 7(D, H) are shown in gray.

bioRxiv preprint doi: https://doi.org/10.1101/2023.03.20.533567; this version posted March 21, 2023. The copyright holder for this preprint (which was not certified by peer review) is the author/funder, who has granted bioRxiv a license to display the preprint in perpetuity. It is made available under aCC-BY 4.0 International license.

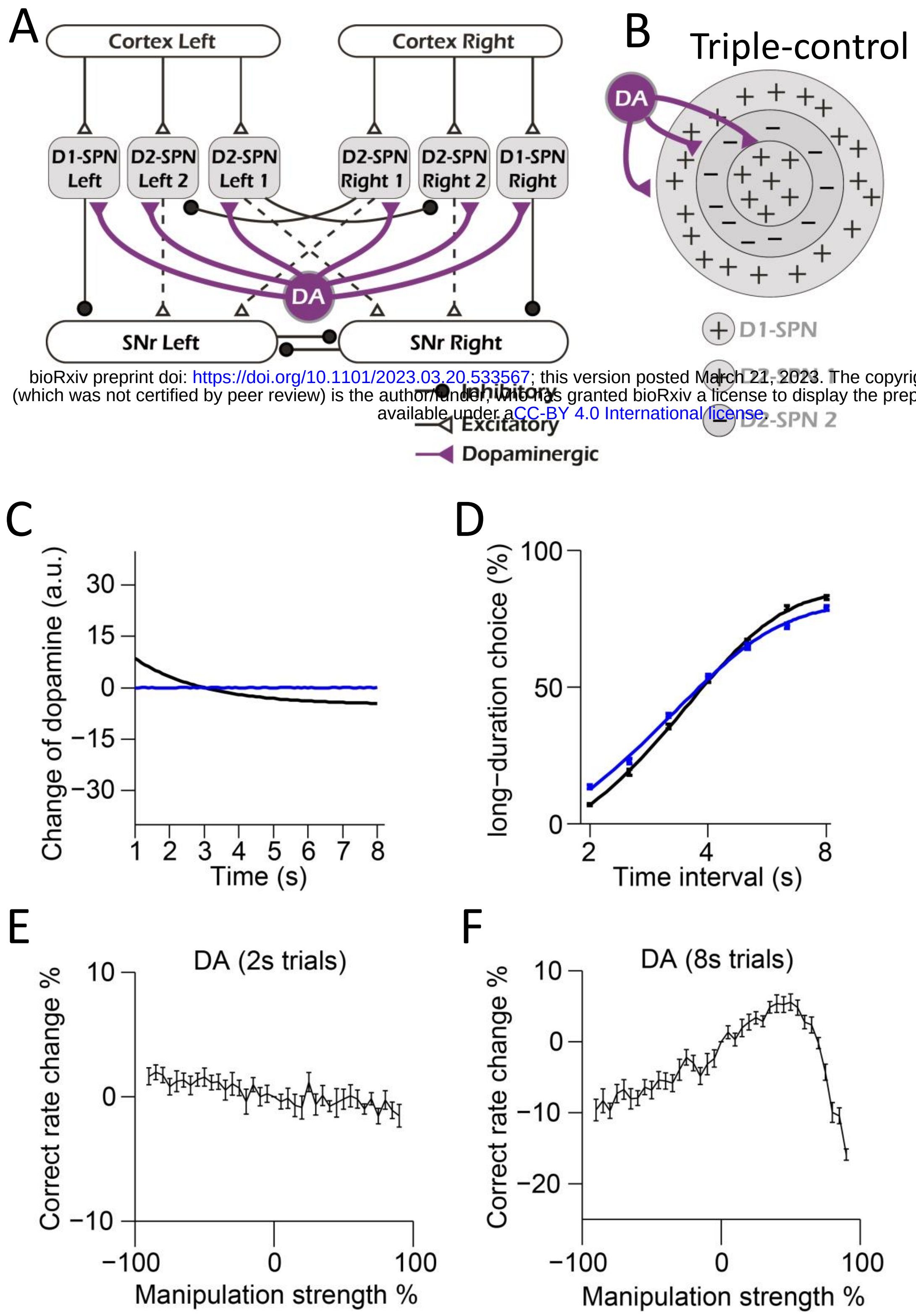

Figure S11. Computational modeling of dopaminergic modulation in the "Triple-control" model. (A) Diagram of Triple-control model with dopaminergic modulation on SPNs. (B) Schematic of center-surround-context receptive field diagram with dopaminergic modulation added for 'Triple-control' model. '+' indicates facilitating effect to selection. '-' indicates inhibitory effect to selection. (C) Simulation of two types of dopamine dynamics (black: decreasing dopamine; blue: constant dopamine with no change) in 8s trials. (D) Psychometric curves corresponding to each dopamine dynamics (n = 10, two-way repeated-measures ANOVA, main effect of ablation, $F_{1,18} = 0.8743$, $p = 0.362$; interaction between trial intervals and ablation, $F_{6,108} = 8.261$, $p < 0.0001$). (E, F) Correct rate change in 2s (E) and 8s trials (F) trials when manipulating dopamine in 'Triple-control' model with different manipulation strengths (n = 10, one-way repeated-measures ANOVA, effect of manipulation strength, 2-s trials: $F_{36,324} = 3.868$, $p < 0.0001$; 8-s trials: $F_{36,324} = 39.98$, $p < 0.0001$).
